# Rapid desensitization through immunoadsorption during cardiopulmonary bypass. A novel method to facilitate human leukocyte antigen incompatible heart transplantation

**DOI:** 10.1177/02676591221151035

**Published:** 2023-01-10

**Authors:** Richard W Issitt, Eamonn Cudworth, Mario Cortina-Borja, Arun Gupta, Delordson Kallon, Richard Crook, Michael Shaw, Alex Robertson, Victor T Tsang, Sophie Henwood, Vivek Muthurangu, Neil J Sebire, Michael Burch, Matthew Fenton

**Affiliations:** 1 Perfusion Department, Great Ormond Street Hospital for Children, London, UK; 2 Institute of Cardiovascular Science, University College London, London, UK; 3 Digital Research, Informatics and Virtual Environment, NIHR Great Ormond Street Biomedical Research Centre, London, UK; 4 Clinical Transplantation Laboratory, 9744 Barts Health NHS Trust, London, UK; 5 Population, Policy and Practice Research and Teaching Department, Great Ormond Street Institute of Child Health, University College London, London, UK; 6 Department of Cardiothoracic Surgery, Great Ormond Street Hospital for Children, London, UK; 7 Department of Cardiothoracic Transplantation, Great Ormond Street Hospital for Children, London, UK; 8 Department of Paediatric Cardiology, Institute of Child Health, University College London, London, UK

**Keywords:** cardiopulmonary bypass, paediatric, transplantation, human leukocyte antigen, immunoadsorption

## Abstract

**Background:**

Anti-human leukocyte antigen (HLA)-antibody production represents a major barrier to heart transplantation, limiting recipient compatibility with potential donors and increasing the risk of complications with poor waiting-list outcomes. Currently there is no consensus to when desensitization should take place, and through what mechanism, meaning that sensitized patients must wait for a compatible donor for many months, if not years. We aimed to determine if intraoperative immunoadsorption could provide a potential desensitization methodology.

**Methods:**

Anti-HLA antibody-containing whole blood was added to a Cardiopulmonary bypass (CPB) circuit set up to mimic a 20 kg patient undergoing heart transplantation. Plasma was separated and diverted to a standalone, secondary immunoadsorption system, with antibody-depleted plasma returned to the CPB circuit. Samples for anti-HLA antibody definition were taken at baseline, when combined with the CPB prime (on bypass), and then every 20 min for the duration of treatment (total 180 min).

**Results:**

A reduction in individual allele median fluorescence intensity (MFI) to below clinically relevant levels (<1000 MFI), and in the majority of cases below the lower positive detection limit (<500 MFI), even in alleles with a baseline MFI >4000 was demonstrated. Reduction occurred in all cases within 120 min, demonstrating efficacy in a time period usual for heart transplantation. Flowcytometric crossmatching of suitable pseudo-donor lymphocytes demonstrated a change from T cell and B cell positive channel shifts to negative, demonstrating a reduction in binding capacity.

**Conclusions:**

Intraoperative immunoadsorption in an *ex* *vivo* setting demonstrates clinically relevant reductions in anti-HLA antibodies within the normal timeframe for heart transplantation. This method represents a potential desensitization technique that could enable sensitized children to accept a donor organ earlier, even in the presence of donor-specific anti-HLA antibodies.

## Introduction

Human leukocyte antigen (HLA) sensitization and the subsequent development of anti-HLA antibodies represents a major barrier to solid organ transplantation, especially in the paediatric setting. In the presence of preformed donor specific antibodies, transplantation is associated with an extremely high incidence of hyperacute rejection and immediate graft failure.^1^ Whilst allosensitization affects between 6-9% of children awaiting a heart transplant, it is significantly greater (35–66%) in those bridged to transplant using ventricular assist devices (VADs).^2–4^ Allosensitization therefore reduces access to donor organs and increases the risk of waiting list mortality and morbidity, as a result of disease progression.^4^ The exact cause of antibody production following VAD implantation remains unknown but is thought likely to be an accumulation of increased immunogenicity of the VAD itself, the requirement for blood product transfusions, and cytokine upregulation during implantation.^5^

Much research is now focused on the management of sensitization using methods that either mechanically filter or bind circulating antibodies, or deplete antibody producing cells, a process known as desensitization.^5^ Several techniques have been proposed, including pre-transplant plasma exchange therapy (PET), double filtration, immunoadsorption (IA) and pre-transplant immunotherapy, although there is no consensus as to the optimal desensitization regime and timing for patients awaiting heart transplantation.^6^ The majority of the literature on desensitization comes from living-donor kidney transplantation, with data for heart transplantation confined to small patient cohorts with limited follow-up durations.^7^ Whilst desensitization may work for an elective transplant with the facility to postpone or delay should conditions not be optimal, this is usually not the case with heart transplantation. The time critical nature of the process demands a different strategy, and being unpredictable, does not lend itself to treating potential recipients regardless of the immanency of transplantation; antibody re-accumulation is likely if no immunosuppression is provided in the pre-transplant setting.^5^ Therefore, a desensitization technique that occurs at the time of transplantation is required.

Previous work has demonstrated the utility of undertaking an intraoperative, targeted PET in a sensitized patient to facilitate a HLA-incompatible (HLAi) bilateral lung transplant.^8^ However, despite the apparent success of this procedure, PET exposes the recipient to multiple donor sources, increasing the risk of transfusion related morbidity.^9^ To mitigate this risk in the paediatric ABO-incompatible (ABOi) heart transplant setting, the methodology of intraoperative IA was developed, which not only significantly reduced blood product transfusions, but also expanded the potential donor pool through increasing the age range of patients eligible for incompatible heart transplantation.^10,11^ Immunoadsorption columns differ in their mechanisms of action dependent on the antibody target. Anti-A/B IA columns utilize ABO-antigen specific ligands, and as a result will not be saturated even at high antibody concentrations.^12^ Conversely, anti-HLA IA columns use a non-specific, synthetic cyclic molecule (such as a GAM-146 peptide) which has a high affinity to human immunoglobulins, especially IgG and immune complexes, and has a binding capacity of approximately 1.2 g of immunoglobulin when fully saturated. For this reason, the two IA columns are not interchangeable, with anti-HLA antibody IA columns requiring frequent column changes or, preferably, a regenerative system.^13^

We therefore investigated the feasibility of integrating into a cardiopulmonary bypass (CPB) circuit, a system where two immunoadsorption columns are utilized in parallel, but with opposite phases of action (one column is actively adsorbing whilst the other is regenerating and reconditioning, ready for use when the first becomes saturated). Using this system, we aimed to determine whether it could reduce anti-HLA antibodies to subclinical levels in an *ex* *vivo* setting, thereby providing a potential intraoperative desensitization methodology to facilitate HLAi heart transplantation in children.

## Materials and methods

### Study design

This laboratory study was approved by the Institutional Review Board as part of a wider study on Antibody Immunoadsorption for Transplantation (19HL02). Anti-HLA antibody-containing whole blood was obtained from NHS Blood and Transplant and detected using Luminex panel bead assay at a United Kingdom Accreditation Service (UKAS) and European Federation for Immunogenetics (EFI) accredited laboratory.

### Ex vivo set up

**Figure 1.**
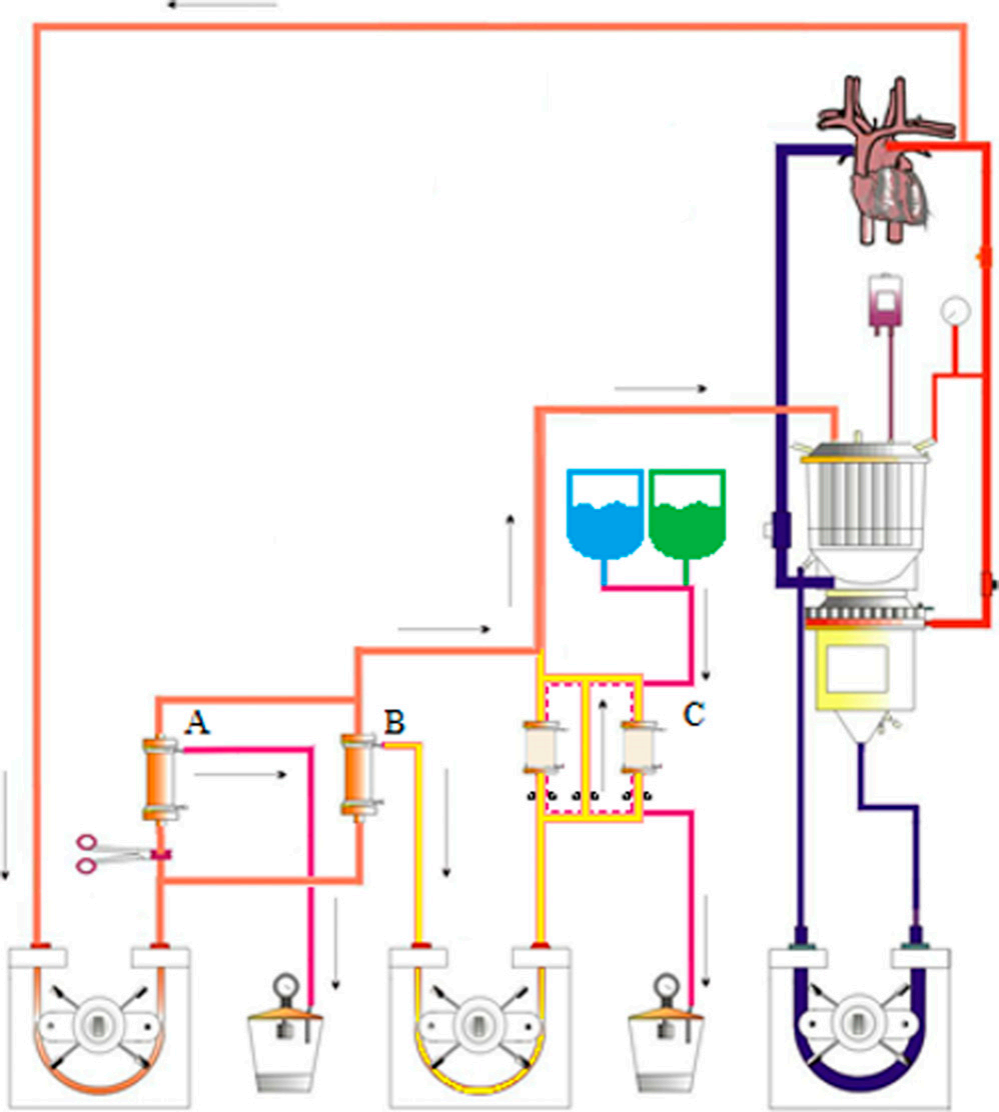
Anti-HLA Immunoadsorption Set-up. Whole blood is pumped, using the ultrafiltration pump, from the arterial limb of the bypass circuit via the plasma separator (b). The haemofilter (a) is clamped from the circuit at this stage, having been used for prebypass ultrafiltration before the start of bypass and later for conventional and modified ultrafiltration. The separated plasma is then pumped through the ADAsorb® Globaffin immunoadsorption system (c) via the immunoadsorption pump. The hemic content from the plasma separator outlet is reconstituted with the antibody-depleted plasma and returned to the circulation via the venous reservoir.

The CPB circuit was set up to mimic a 20 kg patient undergoing heart transplantation and resembled that previously described for ABOi heart transplantation using immunoadsorption.^12^ The circuit consisted of an oxygenator with hard-shell venous reservoir (Capiox RX15-RW30; Terumo, Leuven, Belgium) with a 1/4" x 3/8” arterial-venous loop (LivaNova, London, UK). The tubing set incorporated a hemofiltration circuit controlled via a roller pump, with blood taken from the arterial line and returning to the venous reservoir. A plasma separator (Asahi Kasei OP-08W; LINC Medical Systems Ltd, Leicester, UK) was placed in parallel, via a wye connector, to the haemofilter (HF-06; LivaNova) with the effluent passed via an additional 1/4” roller pump to the immunoadsorption columns (Globaffin®; Fresenius, Bad Homburg, Germany). Adsorption and desorption cycles that control and monitor antibody removal, were undertaken using a secondary system (ADAsorb, Fresenius; Figure 1). Column loading volumes were set to balance adsorption and desorption cycle durations. Following treatment, plasma was returned to the CPB circulation through a 5 µm filter. Arterial flows were set at 2300 ml/min, with blood flow to the plasma separator set at 200 ml/min. Plasma flow was dependent on inlet pressures in the adsorption system and was set between 20 – 30 ml/min accordingly. The circuit was primed with 500 ml of whole human blood, a balanced crystalloid solution (Plasmalyte 148; Baxter Healthcare Ltd, Thetford, UK), and a gelatin colloid (Gelofusine; B. Braun Melsungen AG, Melsungen, Germany), which was anticoagulated with 5,000IU of heparin (Wockhardt UK Ltd, Wrexham, UK) to achieve a haematocrit of 30% mimicking clinical practice. Biochemical compatibility was then attained using sodium bicarbonate as a buffering agent.

### Feasibility study

To determine practical constraints due to the amalgamation of the CPB and secondary adsorption systems, antibody-reduced plasma was returned to the CPB circulation via either the venous return line, or directly into the venous reservoir. Plasma return pressures were then measured to ensure the adsorption system could operate with negative pressures inherent in the CPB circuit. In addition, to simulate clinical conditions as closely as possible, the system was tested with vacuum assisted venous drainage (VAVD) applied to the venous reservoir in the standard manner at both −10mmHg and −20 mmHg. The standard operating range for the adsorption system’s plasma return is −60 to +260 mmHg.

### Anti-HLA antibody detection and definition by Luminex

Based upon a binding capacity per column of 1.2 g immunoglobulin and a total treatment volume of 3-fold the patient’s plasma volume, a total of 4500 ml (75 ml/kg) of plasma was treated. Samples for anti-HLA antibody definition were taken at baseline, when combined with the CPB prime (mimicking bypass initiation), and then every 20 min for the duration of treatment (total 3 h). Anti-HLA antibodies were detected using Luminex single antigen bead assay (Lifecodes Single Antigen Class I/Class II, Immucor GTI Diagnostics Inc, Wisconsin, USA). Where samples did not provide a reliable result with this assay, an alternative single antigen assay was used (LABScreen Single Antigen Class I/Class II, One Lambda Inc, California, USA). Detection and definition of anti-HLA IgG antibodies was undertaken according to manufacturer’s instructions for use. Briefly, samples were treated with EDTA to minimize the “prozone” phenomenon. Where high background Median Fluorescence Intensity (MFI) was detected, samples were treated with serum cleaner (LIFECODES Serum Cleaner, Immucor GTI Diagnostics Inc) when using the Immucor assay or adsorb beads when using the One Lambda assay (Adsorb Out, One Lambda Inc, California, USA).

The Immucor assay was performed as follows: single antigen bead mix and sera were thawed at room temperature, patient sera and control sera were centrifuged for 30 s at 10,000 g to pellet any particulate matter that might be present. Plate wells were pre-wetted using 200 µl of distilled water for 5 min before aspirating. The beads were then thoroughly vortexed for 1 min to ensure even re-suspension, and then 20 µl was added to each of the wells, along with 10 µl of the sample. The plates were then covered and incubated at room temperature for 30 min on a rotating platform set at 200 rpm. The conjugate was prepared by diluting with wash buffer (1:10 dilution) at a concentration of 2.5 µl conjugate to 22.5 µl wash buffer per sample. Following incubation, each well was washed four times with buffer (first wash 100 µl, subsequent washes 250 µl) and then 25 µl of diluted conjugate added to each well. The wells were then covered and incubated for a further 30 min at room temperature on a rotating platform (200 rpm). Finally, the wells were diluted with 130 µl buffer and mixed to re-suspend the beads and read via the Luminex 200 System (Luminex Corporation, Texas, USA). Beads were termed positive if the MFI to lowest ranked antigen (LRA) ratio (calculated by dividing the raw MFI of the bead by the raw MFI of the lowest reacting bead for that locus) was higher than the manufacturer’s predetermined cut-off value, and the raw MFI was greater than 750. Results were adjusted for background by subtracting the negative control sample MFI from the raw MFI for each individual bead and then divided by the relative antigen density for each individual bead (as found in the manufacturer’s lot-specific recording sheet).

The One Lambda assay was performed as follows; single antigen bead mix and sera were thawed at room temperature, patient sera and control sera were centrifuged for 30 s at 10,000 g to pellet any particulate matter that might be present. Plate wells were pre-wetted using 200 µl of wash buffer for 10 min before aspirating. The beads were then thoroughly vortexed for 1 min to ensure even re-suspension, and then 2 µl was added to each of the wells, along with 10 µl of the sample. The plates were then covered and incubated at room temperature for 30 min in the dark on a rotating platform set at 200 rpm. The conjugate was prepared by diluting with wash buffer (1:100 dilution) at a concentration of 1 µl conjugate to 99 µl wash buffer per sample. Following incubation, each well was washed three times with 100 µl wash buffer and then 100 µl of diluted conjugate added to each well. The wells were then covered and incubated for a further 30 min at room temperature in the dark on a rotating platform (200 rpm). Finally, the wells were washed three times with 200 µl buffer and re-suspended in 80 μl phosphate buffered saline, samples were then read via the Luminex 200 System. Results were adjusted for background by subtracting the negative control bead MFI from the raw MFI for each individual bead.

Antibody specificity was grouped based upon background adjusted MFI levels as follows: MFI >2000 (Positive), 1000 < MFI >2000 (Weak positive), 1000 < MFI >500 (Very Weak positive), MFI <500 (Negative).

### Pseudo-Donor flowcytometric crossmatching

Anti-HLA antibody specificities determined using Luminex testing were cross-referenced with HLA typing data from transplant donors who donated to patients treated at Barts Health NHS Trust, to identify lymphocytes which expressed antigen corresponding to anti-HLA antibodies detected. Where matches existed, stored lymphocytes from these donors were selected as “pseudo-donor organs” and were used to provide flowcytometric crossmatches for the experimental samples. Samples were treated with a standard three-colour technique, and with negative and positive control sera, using a FACSLyric Flow Cytometer (BD Biosciences, California, USA). Briefly, experimental samples were centrifuged at 13,000 rpm for 5 min, whilst donor lymphocytes were centrifuged at 740 g for 5 min. Following discarding of the supernatant, lymphocyte concentration was adjusted to 4.0 × 10^6^ cells/ml. Control or test sera was added to lymphocytes in equal volumes (25 µl) and vortexed before incubation at 22°C for 30 min. Samples were centrifuged and washed twice (740 g and 2 ml Flow Diluent) before incubating with 5 µl of diluted anti-Human IgG and 5 µl anti-CD3/CD19 conjugates for 25 min in the dark at 22°C. Samples were washed again and re-suspended in 280 µl of Flow Diluent before loading on the FACSLyric platform. The Mean Channel Shift (MCS) was calculated by subtracting the mean channel of the negative control from the mean channel of the serum sample. A MCS >80 was deemed positive. Strong positive controls were in the range of 100–1000 MCS, whilst the weak positive control was 40–200 MCS. Interactions between lymphocytes and donated blood were also qualified using Luminex single antigen assay as described above.

### Statistical modeling

Data analyses was performed using the R language and environment for statistical computing, version 4.1.0 (R Foundation for Statistical Computing) using the tidyverse suite of packages (v1.3.1).^14,15^ Scatterplots of MFI data from Luminex assay against sampling time were summarized with a non-parametric smoothing curve (Locally Weighted Scatterplot Smoothing; Lowess) fitted at the alleleic level, providing a continuous estimate over discrete time points. Estimations of immunoadsorption treatment duration were undertaken using both centile curves and smoothing spline prediction. Centile curves from the data were based on semiparametric regression within the generalized additive models of location, scale and shape (GAMLSS) using the gamlss package.^16,17^ We chose the four parameter Sinh-Arcsinh (SHASH) model as an adequate representation of the asymmetry and differential tail weight present in the distributions of MFI.^18^ We fitted linear predictions based on natural penalized cubic splines for each of the SHASH model’s parameters using the splines package.^19^ Model selection depending on the number of degrees of freedom as indicators of model complexity was undertaken using a grid-search technique to minimize the Bayesian information criterion (BIC).^20^ Model normality and homoscedasticity were confirmed using residual plots. Smoothing spline predictions with cross validation to determine the degree of smoothness were obtained for treatment duration estimation based upon baseline MFI and required percentage reduction. The time point at which the smoothed curve crossed an MFI threshold of 500, or reached the required percentage reduction, was calculated using a fine grid-search, and estimated marginal means for treatment duration were calculated for the 95% confidence intervals using the effects package.^21^

## Results

### Feasibility study

Plasma return pressures from the adsorption system to the CPB circuit was tested at both the venous reservoir port and directly into the venous return line itself. It was noted that in test conditions, the venous reservoir provided a negative pressure of −20 mmHg (which corresponded to the difference in height of the two systems: the venous reservoir sitting lower than the adsorption system). By comparison, plasma flow into the venous return line increased the negative pressure to −35 mmHg. Simulating clinical conditions where VAVD is required (and applied to the venous reservoir vent port) at both −10 mmHg and −20 mmHg demonstrated that there was a consistent 10 mmHg difference in plasma return pressures in favour of the venous reservoir (Table 1). All conditions provided a plasma return pressure above the minimum operating pressure of the adsorption system (−60mmHg).

**Table 1.** Feasibility data.

	Plasma return pressures (mmHg)	
VAVD (mmHg)	Venous line	Venous reservoir
0	−35	−20
−10	−40	−30
−20	−50	−40

Plasma-return pressures measured on the ADAsorb system. VAVD; vacuum assisted venous drainage. VAVD set to stated levels and pressures measured using electronic pressure monitoring of the heart-lung machine at the stated positions.

### Anti-HLA antibody immunoadsorption

**Figure 2.**
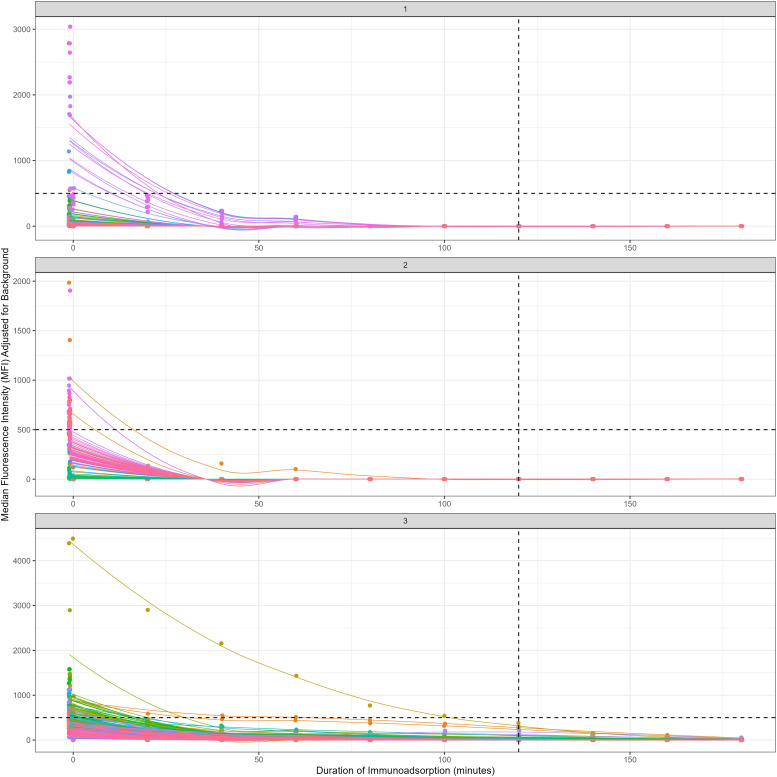
Data from the HLA-IA *ex vivo* simulation showing the reduction over time of anti-HLA antibody as measured by Luminex assay. Lines produced by lowess smoothing of the individual allele result. The horizontal black line represents the clinical importance threshold of 500. The vertical black line represents the typical time from initiation of CPB to organ reperfusion (120 min). All samples were reduced below the threshold within the time frame described.

Donated blood units showed a baseline cumulative MFI between 45,106 and 112,791, encompassing both Class I and Class II subgroups (Table 2). Following addition of the CPB prime solutions, MFI dropped by a mean of 77% due to haemodilution. Luminex results demonstrated a consistent reduction in MFI across the experiments, with all alleles of both Class I and II antibodies passing the lower clinically relevant limit (MFI <500) within 120 min of treatment. By 180 min, 86% of alleles were undetectable, with the remaining 14% having a mean MFI of 16 (Figure 2).

**Table 2.** Donated-blood details.

Donated blood unit	Cumulative MFI	Selected alleles (MFI)
1	45,106	DQA1*01:03 (3038); DQA1*01:02 (2785)
		DQB1*06:04 (2643); DQA1*03:02 (2267)
2	53,909	A*29:02 (1984); DQA1*01:02 (1904)
		A*29:01 (1404)
3	112,791	B*15:02 (4387); B*40:01 (2895)
		B*45:01 (1581); A*29:02 (1208)
		DQA1*02:01DQB1*03:01 (1131); DPA1*04:01DPB1*28:01 (1038)

All blood units were obtained from NHS Blood and Transplant. All MFI values were obtained using Luminex, solid phase assay. Selected alleles represent those alleles with the highest MFI in each unit.

### Pseudo-Donor crossmatch

**Figure 3.**
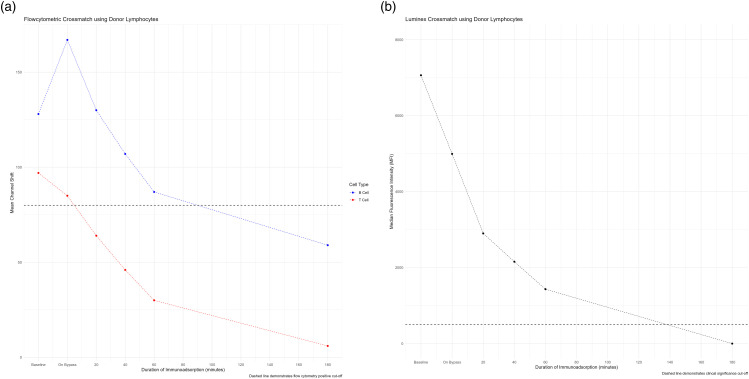
Donor lymphocytes were matched to alleles determined by Luminex testing and then tested using flowcytometry (**a**) to establish T cell and B cell antibody binding. A mean channel shift of >80 (horizontal black line) was considered positive. Both T cell and B cell responses were negative following treatment. Samples were also tested using Luminex for specific DSA (**b**). A cumulative MFI >7000 was reduced to 0 within the experimental time period.

Previous transplant donor lymphocytes were crossmatched to find donor-specific matches to the antibodies in the experimental whole-blood units. Due to finite quantities of donor lymphocytes, only baseline, initial bypass prime, first three and last treatment cycles were tested. Units one and two were matched for donor-specific antibodies but had low cumulative MFI levels and were negative for T cell and B cell binding (Table 3). The final unit demonstrated a higher cumulative MFI (7064) with positive MCS in both T cell and B cell testing. There was an increase from baseline in B cell MCS to 167 in the initial bypass prime sample, but this resolved and was weak positive (MCS 87) at the end of the third treatment cycle and negative (MCS 59) following 180 min of treatment. T cell binding decreased in a linear fashion and was weak positive (MCS 64) following the first treatment cycle and negative (MCS 46) following the second treatment cycle. Following 180 min of treatment, T cell binding remained negative with a MCS of 6 (Figure 3(a)).

**Table 3.** Pseudo-Donor crossmatch.

Pseudo-patient	Cumulative MFI	Details
1	3086	DQ6 (3086)
		T cell -ve; B cell -ve
2	3888	A29 (1984); DQ6 (1904)
		T cell -ve; B cell -ve
3	7064	B75 (4570); B35 (932); Cw8 (848); DPB1*04:02 (714)
		T cell + ve (97); B cell + ve (128)

Donor specific antibodies are given with MFI in parentheses. Flowcytometric results are given as mean channel shift in parentheses where the result is positive.

Luminex testing against donor lymphocytes demonstrated a steady decrease in MFI with an 80% reduction achieved following the third treatment cycle (cumulative MFI 1432; Figure 3(b)). The final sample, taken after 180 min of treatment demonstrated no detectable antibodies.

### Estimation of immunoadsorption treatment duration

**Figure 4.**
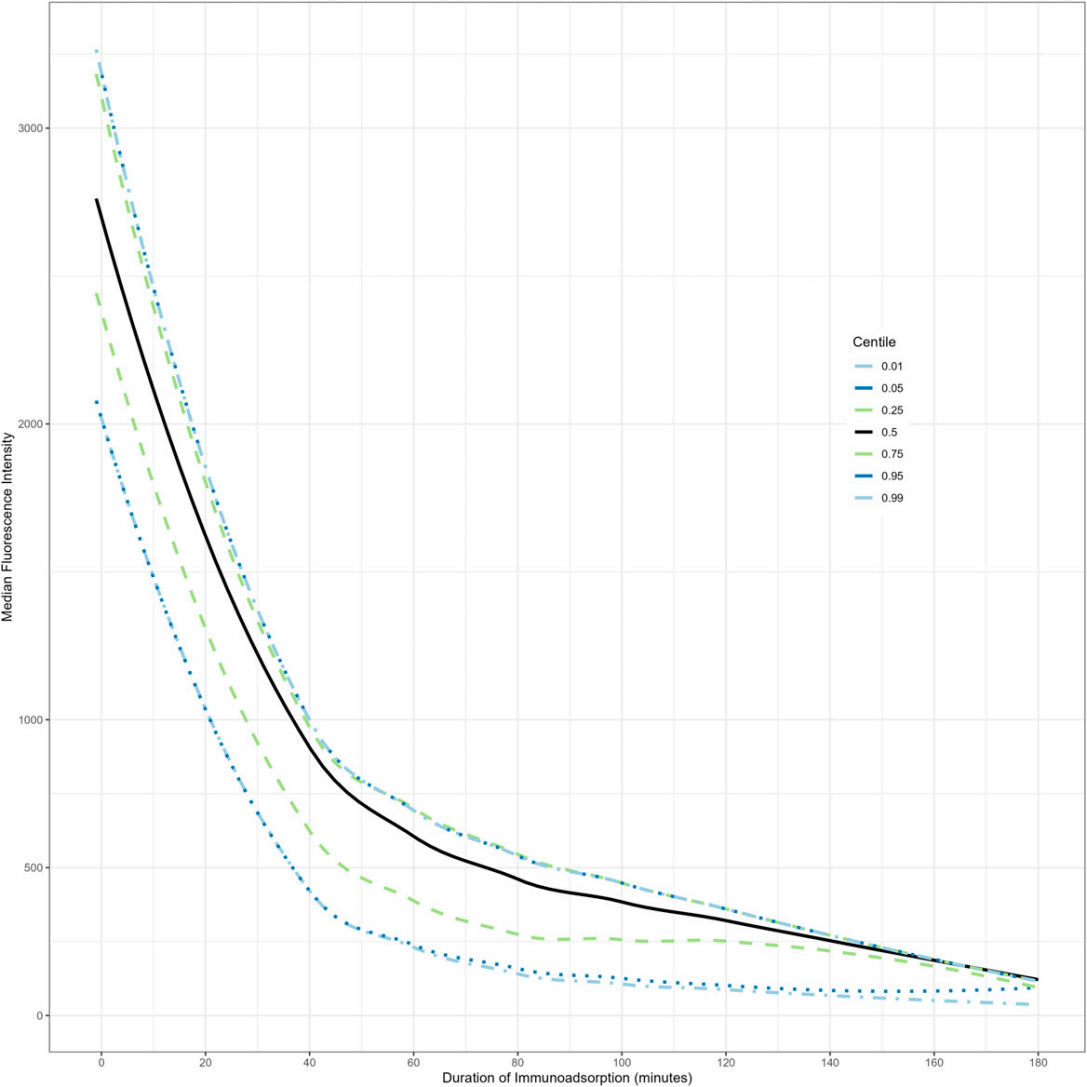
Quantile estimation of anti-HLA antibody immunoadsorption using a GAMLSS model with SHASH distributions. Model demonstrates that all centiles are brought under the 500 MFI threshold within 120 min.

In order to model the continuous nature of immunoadsorption over discrete sampling timepoints, centile plots were created using the GAMLSS model with optimal BIC. The final model was defined by a penalized spline of immunoadsorption time with 9 degrees of freedom for central location (μ), and a penalized spline of immunoadsorption time with 2 degrees of freedom for scale (σ), with the individual antibody’s genetic region as an interaction term. Distributional asymmetry (ν) and tail weight (τ) were estimated without being conditional on timepoints (model residuals are shown in Supplementary Data). The resultant centile plot demonstrated all antibodies decreasing below the clinical MFI threshold of 500 after 100 min of treatment (Figure 4).

**Figure 5.**
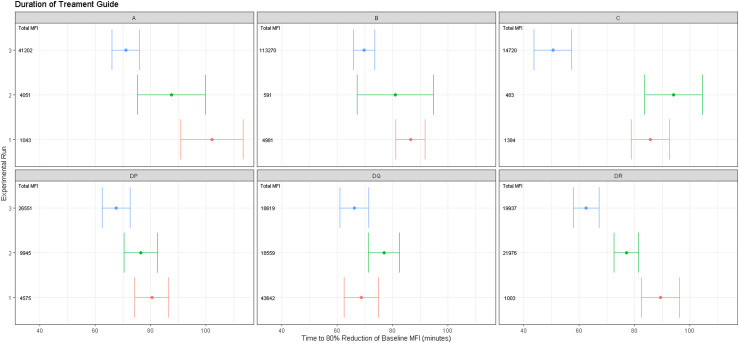
Visualization of the time taken for a required reduction in MFI (80%; based on a previous patient report that was unable to undergo transplantation because of HLA incompatibility). Data are split by alleleic chromosomal region. Dot represents the median time to reduction with the error bars illustrating the 95% confidence intervals calculated using a cubic spline model. Intention is to give the clinician a clear visual guide to how long IA treatment is required to assess feasibility of completing HLA-IA process, and therefore whether patient is suitable for HLAi transplantation.

To account for alleles in the population that hadn’t been observed in the donated blood used in the experiment, as well as varying antibody concentrations, smooth spline predictions were made of MFI across a fine time-grid for the individual genetic regions that encode anti-HLA antibodies (Class I – A, B, C; Class II – DP, DQ, DR) seen in these experiments. Based upon a hypothetical reduction, it was then possible to determine the median time (and its 95% confidence intervals, computed directly from the model’s prediction) required to reach that reduction across a range of cumulative MFI values, all of which occurred within 120 min of treatment (Figure 5).

## Discussion

Desensitization of paediatric patients with donor-specific anti-HLA antibodies awaiting transplantation, may be an important factor in maximizing donor organ compatibility, minimizing the risk of waiting-list mortality, as well as the potential for hyperacute rejection following transplantation.^22^ However, there is no consensus as to who should undergo desensitization, by what method and when, with different strategies showing mixed results.^4^ Recent work has shown that pre-transplantation desensitization is highly variable in patients, but appears to be successful, although larger cohort studies with longer follow-up durations are required.^23^ Desensitization using immunoadsorption has been undertaken in combination with intravenous immunoglobulin in the pre-transplant period, and has shown anti-HLA antibody reductions of 50–70%, but with evidence of rebound to pre-treatment levels within 1 week of therapy.^24^ Moreover, weekly treatment is required, placing a burden not only on the potential recipient but also on the health teams treating them. Furthermore, because of the requirement for patients to support their own circulation during this treatment, and the anticoagulation required, hypocalcaemia and instability are potential risks, and immunoadsorption is therefore undertaken slowly to minimize these risks.^25^ Previous work has demonstrated the efficacy of anti-HLA antibody desensitization in the intraoperative period using PET in the setting of paediatric lung transplantation.^8^ However, PET is associated with a number of issues, primarily resulting from the large volume of donated blood products required to achieve adequate dilution.^9^ To alleviate these side effects in the context of ABOi heart transplantation, we developed the method of intraoperative immunoadsorption, which provides equivalent antibody removal to plasma-exchange.^10,11^ Due to the nature of CPB, the recipient’s circulation can be managed and maintained, regardless of the rate and volume of plasma treated, without the issues of hypocalcaemia posing a problem to cardiac function.^12^

This *ex* *vivo* study has demonstrated the ability to potentially replicate that success in desensitization in the context of the HLA system, offering a viable method for routine HLAi heart transplantation in children, and expanding the potential donor pool for patients whose sensitization is limiting access to donor organs.

### Integrating Anti-HLA antibody immunoadsorption

The success of using immunoadsorption for ABOi heart transplantation is partly reliant on the (relative) simplicity of the system, and the fact that, even at very high anti-A/B isohaemagglutinin concentrations, the immunoadsorption column will not saturate, as it contains specific A/B-antigens as target ligands.^12^ Immunoadsorption columns that remove anti-HLA antibodies are designed to remove all immunoglobulins and immune complexes using synthetic peptides. The super-locus for HLA-antigens, held on Chromosome 6, represents 3.78mb of genomic data (far larger than the 18kb genomic region for the ABO system), and is the most polymorphic genetic region in the human genome with 34,422 alleles identified to date.^26^ Due to the vast quantities and variations of HLA-antigens, and therefore of anti-HLA antibodies, a simple, single antigen-targeted immunoadsorption column (like the anti-A/B columns used above) is impractical. Equally, a single universal column would quickly become saturated, leading to pressure overload and risk of component rupture which, in turn, increases the risk of patient complications due to embolic events.^27,28^ Therefore, a more complex solution is required.

We tested a two-column regenerative immunoadsorption system, adjunct to the CPB circuitry. This system utilizes opposite phases of action for the columns, adsorbing antibody on one whilst the other regenerates and reconditions, ready for use when the first becomes saturated, managed independently of the CPB circuit. A major barrier to incorporating two disparate technologies is the potential incompatibility of their operating limitations, and the additional complexity to manage them safely. In this case it was potential for the plasma return pressure (from the adsorption machine into the CPB circuit) to fall below the lower limits of operation on the adsorption system. Ergo, we tested the system on different access and return ports on the CPB circuit. We observed a less negative pressure when using the venous reservoir compared to the venous return line. This difference is likely due to the Venturi effect caused by fluid flowing past the plasma return port. However, using either port, even with the added suction of a VAVD device, negative pressures remained above the minimum threshold that the immunoadsorption system could operate on. Therefore, we demonstrated that the two separate systems can be utilized in parallel, with the CPB circuit feeding plasma at sufficient flow to facilitate anti-HLA antibody immunoadsorption under clinically relevant conditions.

### Immunoadsorption reduces anti-HLA antibodies in an ex vivo model

In order to assess the efficacy of anti-HLA antibody immunoadsorption in a CPB circuit, we used two standardized methods of measurement for cross-validation: solid phase, single antigen assay (Luminex) and flowcytometric crossmatching using donor lymphocytes. To make this clinically relevant and be a realistic method of desensitization, the timeframe chosen to evaluate efficacy was based on the mean CPB time to reperfusion of the donor organ in both primary and redo-sternotomy transplant recipients (120 and 180 min respectively in this institution). Any prolongation of CPB beyond this timeframe has been shown to be extremely detrimental and associated with increased neurological injury and disruption of normal haemostatic integrity.^29^ Furthermore, the size of the CPB circuit and priming volumes were chosen to represent a sensitized paediatric patient undergoing heart transplantation (based upon a typical 20 kg, VAD-supported patient at the institution), and the levels of antibodies (cumulative MFI >5000) classed as high risk for hyperacute rejection under UK guidelines.^30^ We observed an exponential decay in the amount of antibody measured with the solid phase Luminex assay, to levels considered qualitatively negative (MFI <500). There were no differences between Class I or Class II anti-HLA antibodies in rate of removal, and sub-clinical values were observed in all experiments, within the timeframe specified (Figure 2). By using a smooth spline model, it was also possible to demonstrate that the time for a predetermined percentage reduction was similar regardless of the alleleic subtype and baseline MFI level.

As single antigen assays are considered semiquantitative (and indeed the FDA only approve this method for qualitative analysis), we also used flowcytometric crossmatching, which is sensitive to both complement and non-complement binding of Class I and Class II antibodies, for cross-validation.^31^ Using lymphocytes of previous donors, we corroborated the exponential decay observed in the Luminex assay, and were able to demonstrate a positive-to-negative shifting of antibody binding within the experimental timeframe, although it should be noted that because of limited lymphocyte availability, not all time points were tested.

## Conclusions

This study was designed to exceed the current limits of clinical practice to demonstrate the efficacy of intraoperative immunoadsorption as a potential desensitization technique for paediatric patients at high risk of hyperacute rejection awaiting transplantation. Such a method would potentially expand the donor pool of organs, as well as decrease the amount of time on the waiting list. We have demonstrated that in an *ex* *vivo* laboratory setting, as well as by using donor lymphocytes, the described method can successfully remove anti-HLA antibodies to sub-clinical levels, with a corresponding reduction in T cell and B cell binding capacity, all within the normal time frame associated with heart transplantation. Clinical implementation of this system is now planned to assess the long-term outcomes of this novel technique, and the impact it would have on heart transplantation in children.

## Supplemental Material

Supplemental Material - Rapid desensitization through immunoadsorption during cardiopulmonary bypass. A novel method to facilitate human leukocyte antigen incompatible heart transplantation Supplemental Material for Rapid desensitization through immunoadsorption during cardiopulmonary bypass. A novel method to facilitate human leukocyte antigen incompatible heart transplantation by Richard W Issitt, Eamonn Cudworth, Mario Cortina-Borja, Arun Gupta, Delordson Kallon, Richard Crook, Michael Shaw, Alex Robertson, Victor T Tsang, Sophie Henwood^7^, Vivek Muthurangu, Neil J Sebire, Michael Burch and Matthew Fenton in Perfusion
