# Supplementary material for: Rapid desensitization through immunoadsorption during cardiopulmonary bypass. A novel method to facilitate human leukocyte antigen incompatible heart transplantation
Source: Perfusion. 2023 Jan 10;39(3):543–54. doi: 10.1177/02676591221151035 (PMC10943618; doi:10.1177/02676591221151035)

Supplementary Data

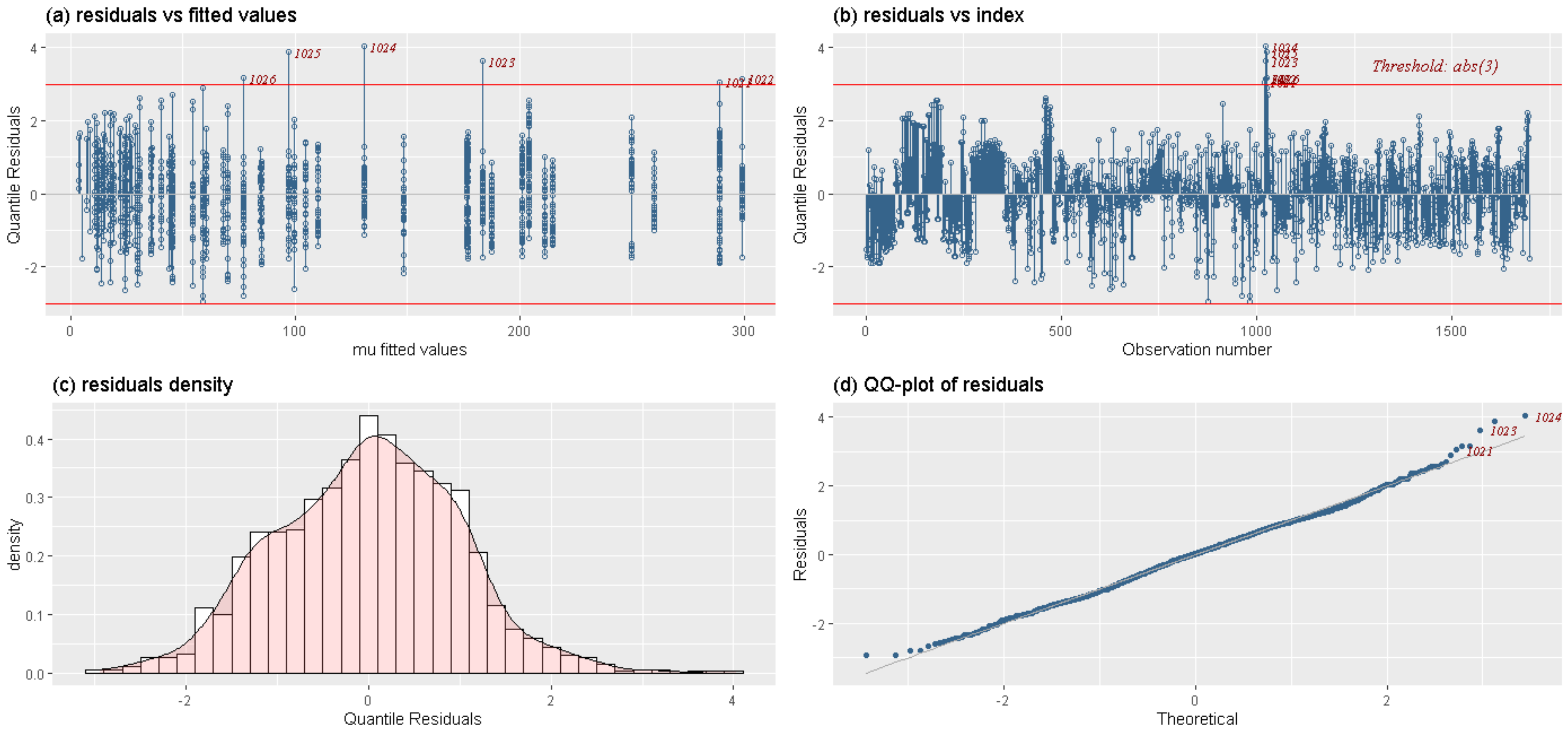

Supplement: Supplemental Material - Rapid desensitization through immunoadsorption during cardiopulmonary bypass. A novel method to facilitate human leukocyte antigen incompatible heart transplantation [file sj-pdf-1-prf-10.1177_02676591221151035.pdf]
